# Citizen Science Approach to Home Radon Testing, Environmental Health Literacy and Efficacy

**DOI:** 10.5334/cstp.472

**Published:** 2022-06-02

**Authors:** STACY STANIFER, ANNA GOODMAN HOOVER, KATHY RADEMACHER, MARY KAY RAYENS, WILLIAM HANEBERG, ELLEN J. HAHN

**Affiliations:** University of Kentucky, US

**Keywords:** citizen science, environmental health, self-efficacy, health literacy, radon

## Abstract

Exposure to radon is a leading cause of lung cancer worldwide. However, few test their homes for radon. There is a need to increase access to radon testing and decrease radon exposure. This longitudinal, mixed-methods study using a citizen science approach recruited and trained a convenience sample of 60 non-scientist homeowners from four rural Kentucky counties to test their homes for radon using a low-cost continuous radon detector, report back findings, and participate in a focus group to assess their testing experience. The aim was to evaluate changes in environmental health literacy (EHL) and efficacy over time. Participants completed online surveys at baseline, post-testing, and 4–5 months later to evaluate EHL, response efficacy, health information efficacy, and self-efficacy related to radon testing and mitigation. Mixed modeling for repeated measures evaluated changes over time. Citizen scientists reported a significant increase in EHL, health information efficacy, and radon testing self-efficacy over time. While there was a significant increase in citizen scientists’ confidence in their perceived ability to contact a radon mitigation professional, there was no change over time in citizen scientists’ beliefs that radon mitigation would reduce the threat of radon exposure, nor was there a change in their capacity to hire a radon mitigation professional. Further research is needed to understand the role of citizen science in home radon mitigation.

## BACKGROUND

Radon exposure is a leading cause of lung cancer worldwide, accounting for approximately 3–14% of all lung cancers (World Health Organization [WHO] 2009). In the US, lung cancer is the second most commonly diagnosed cancer and the leading cause of cancer mortality in both men and women, yet the disease remains highly preventable (American Cancer Society [ACS] 2021). Tobacco smoke and radon exposure cause most lung cancer cases (American Lung Association [ALA] 2021). While tobacco use is the leading cause of lung cancer, radon, a naturally occurring radioactive gas, is the second leading cause, responsible for approximately 21,000 radon-induced lung cancer deaths annually in the US (Environmental Protection Agency [EPA] 2021). While non-smokers are at risk for lung cancer from radon exposure, for those who smoke or are exposed to tobacco smoke, the harmful effects of radon are even greater. For example, at 4.0 picocuries per liter of air (pCi/L) of radon exposure over a lifetime, approximately 62 out 1,000 smokers and 7 out 1,000 never-smokers could develop lung cancer (EPA 2021).

Homes are a major source of radon exposure as the radioactive gas can enter and become trapped inside. Because radon gas is odorless, colorless, and tasteless, testing one’s home for radon is necessary to determine exposure risk. In 2005, the U.S. Surgeon General released a national health advisory on radon urging all Americans to test their home for radon and take action to reduce levels when radon levels are ≥ 4.0 pCi/L (US Department of Health and Human Services, 2005). Despite the warning, few Americans test their homes for radon. A telephone survey of radon awareness, testing, and remediation was conducted with 1,209 randomly selected New York residents. Half the sample lived in a high-radon county and the other half lived in a low-radon county based on an EPA map of radon zones. Findings revealed that while 82% reported having heard of radon, only 15% had tested their home (Wang et al. 2000). A recent analysis of 20 years of observed home radon values in Kentucky reported an annual residential radon testing rate of only 13.4 per 10,000 households (Stanifer et al. 2021). Furthermore, many US residents are unfamiliar with radon (Nissen et al. 2012) and its health effects (Duckworth et al. 2002), and few view radon as an immediate health risk in their homes (Wang et al 1999; Duckworth et al. 2002). The relatively low public knowledge of radon and low percentage of Americans who have tested their homes for radon necessitates renewed efforts and methods for increasing the public’s knowledge of radon and home radon testing and mitigation.

Citizen science, a research approach by which the public addresses community concerns through active participation with scientists during the research process, has demonstrated its ability to prompt action (King et al. 2021) and increase knowledge and skills (Peter et al. 2021). Citizen science approaches differ from traditional research methods in that non-scientists are recruited to collect and analyze their own data and are trained in rigorous scientific methods as part of the research process (Booth al. 2020; Odunitan-Wayas 2020). Another characteristic of the citizen science approach is the report back, meaning the non-scientists are engaged in reviewing and synthesizing the results and reporting back the findings to local stakeholders (King et al. 2021). Report back may occur using different modes of communication and various target audiences. For example, Odunitan-Wayas (2020) recruited non-scientists to use a mobile app to take photos and use audio narratives to evaluate barriers and promoters of physical activity in a low-income South African community, and the citizen scientists facilitated a workshop/community meeting to review and prioritize their findings with community members and suggest potential solutions. Citizen science approaches, unlike traditional research, may be referred to as participatory action research because these approaches are often designed to contribute to positive change, engage participants in realistic solutions, and move the research to action (King et al. 2021; Odunitan-Wayas et al. 2020). A recent systematic review of citizen science contributions to radon research included eight past or ongoing citizen science initiatives from five different countries (including the study reported here) (Martell et al. 2021). The review concluded citizen science approaches have generated new knowledge and understanding of radon in the scientific community while also increasing home radon testing and, to a lesser degree, mitigation in the general population. The study reported here contributes to the body of knowledge, particularly our understanding of citizen science contributions to radon research in rural communities where access to radon testing and mitigation is often challenging.

Having the necessary public knowledge and information to protect human health from environmental hazards, like radon, requires environmental health literacy (EHL) (Gray 2018). EHL is the ability to recognize a specific environmental health hazard, understand the source(s) of the hazard, apply that understanding to taking preventive health action, assess personal risk of exposure, evaluate ways of mitigating risk, and create a plan to mitigate the risk (Finn and O’Fallon 2017). Most EHL studies measure knowledge, attitudes, and beliefs about one or more specific toxicants (e.g., radon) to identify levels of EHL (Gray 2018). EHL-building interventions and methods, including citizen science approaches, draw from social science fields like health communication, health literacy, and risk and participatory communication (Hoover 2019). Finn and O’Fallon (2017) suggest EHL assessments be tailored to the topic and population. Recent examples of EHL interventions have improved the diagnosis of air pollution information needs (Ramirez et al. 2019) and have improved environmental health knowledge by developing and adapting a mobile app (Dellinger et al. 2019). In addition, citizen science approaches using environmental sampling and Photovoice with Appalachian and urban youths have built knowledge, efficacy, and engagement (Cardarelli et al. 2021), while research in California engaged high school students in community air monitoring (Madrigal et al. 2020). A goal of EHL is to foster greater understanding of environmental health risks among individuals, which in turn prompts action to reduce risk (Finn and O’Fallon 2017). Indeed, those with higher self-efficacy in radon testing and mitigation were more likely than those with lower self-efficacy to take action to test and remediate for radon (Hahn et al. 2019).

Given that citizen science approaches often empower people to take action and adapt their own environmental health behaviors, we launched Radon on the RADAR (Residents Acting to Detect and Alleviate Radon), a community-engaged, citizen science research collaboration to promote home radon testing in four rural counties of Kentucky. We trained 60 non-scientist community residents as citizen scientists to test their homes for radon and report back the findings. We engaged them in a focus group to evaluate their experience. We evaluated changes in EHL and efficacy via an online survey administered at baseline, post-radon testing, and at 4–5 months after implementing a citizen science approach. We hypothesized that EHL, response efficacy, health information efficacy, and self-efficacy related to radon testing and mitigation would increase over time.

## MATERIALS AND METHODS

### DESIGN AND SAMPLE

This is a longitudinal, mixed-methods study. Data reported in this paper were collected from each citizen scientist participant at three repeated timepoints. We used convenience sampling to recruit 15 citizen scientists from each of four rural counties in Kentucky. Inclusion criteria were: homeowner, at least 21 years old, no plans to move in the next two years, willing to test their home for radon, daily access to the internet, and able to read and understand English.

**Figure 1:**
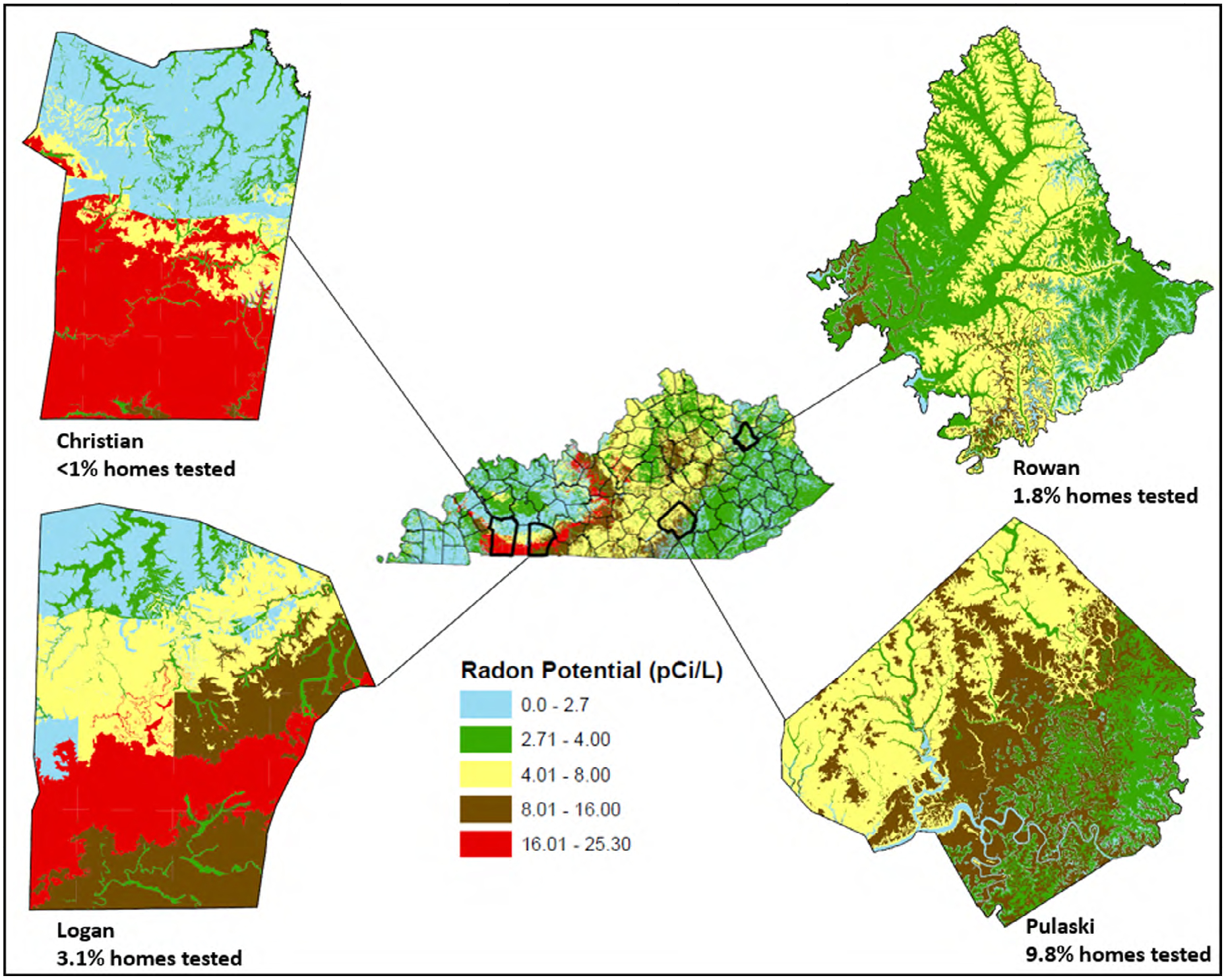
Radon risk potential by study county.

The four rural counties were selected based on geology/radon risk potential and were matched on county-level median income and population size. We chose two geologically similar counties in western Kentucky (Christian and Logan counties), along the edge of the Illinois Basin and transected by a band of Mississippian limestone with extremely high radon risk potential (16.01–25.30 pCi/L, the standard U.S. metric for radon) and intense karst (cave) potential (see Figure 1). We chose the other two counties (Rowan and Pulaski), also geologically similar to each other, along the boundary between the Mississippian and Cumberland Plateaus and underlain by Mississippian and Pennsylvanian limestone and clastic bedrock, with lower karst and radon risk potential (see Figure 1) (Haneberg et al. 2020). Rowan and Pulaski counties were chosen from among the rural counties with lower radon risk potential because they matched the two high radon potential counties on population size and median income. Christian and Pulaski had population sizes of 73,995 and 63,063, while the populations of Logan and Rowan were 26,835 and 23,333, respectively (US Census Bureau 2010); all four counties had median household incomes ranging from $34,000 to $39,000 (US Census Bureau 2017).

### STUDY COMPONENTS AND PROCEDURES

This study was approved by the Institutional Review Board of a large public university located in the southeastern United States. We established partnerships with Area Health Education Centers (AHEC) in two study counties (Rowan and Pulaski), an Area Development District office in Christian County, and a library in Logan County to lead recruitment, consent, and enrollment efforts in each of the four study counties. Each recruiting site has a Site Principal Investigator (PI) and a Site Coordinator who advertised the study using social media, local newspapers and radio, study flyers, and listservs. Recruitment flyers included the statement, “Want to know if you are exposed to radon in your home?” Interested individuals completed a brief online screening survey to determine eligibility. Individuals who were eligible and willing to participate completed a contact form, and Site Coordinators scheduled appointments to explain the study and get consent from interested respondents. Due to the COVID-19 pandemic, research activities were restricted to online/phone interactions. We used REDCap (Research Electronic Data Capture; Harris et al. 2009) electronic data capture tools hosted at the University of Kentucky to develop, collect and manage an electronic consent process. REDCap is a secure, web-based application designed to support data capture for research studies, providing: 1) an intuitive interface for validated data entry; 2) audit trails for tracking data manipulation and export procedures; 3) automated exposure procedures for seamless data downloads to common statistical packages; and 4) procedures for importing data from external sources (Harris et al. 2009). Site Coordinators obtained consent from participants using REDCap over the phone or via Zoom.

**Figure 2:**
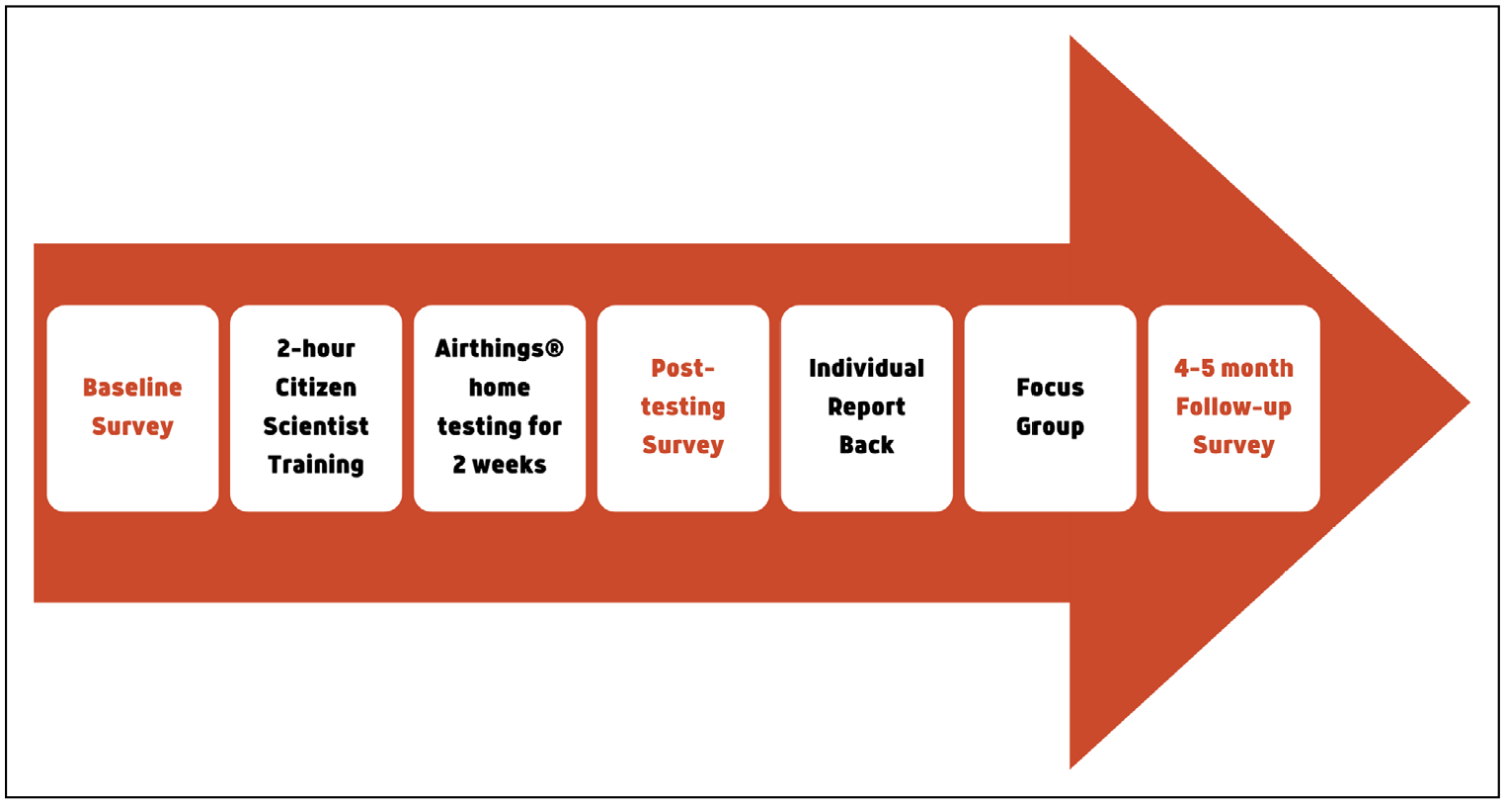
Data collection, training, radon testing, and report back time points.

Following informed consent, participants completed a brief online survey to assess baseline EHL and efficacy, as well as demographics. We scheduled a 2-hour group training session via Zoom, and trained participants as citizen scientists (see citizen science training details below). Upon completion of the training, each citizen scientist received a certificate of completion and joined our research team to test their home for radon over a 2-week period using a low-cost Airthings® Corentium Home Radon Detector (see testing details below). With each participant, we provided personalized email report back of their daily and long-term average radon values; if participants had high radon levels, we invited them to a telephone conversation to assess their concerns, interest in radon mitigation, and next steps (see report back details below). For those with radon values ≥4.0 pCi/L, we provided a voucher to cover 30% of the cost of radon mitigation up to $600. After radon testing and report back, we invited citizen scientists to take part in a focus group to assess their experience of testing their home for radon. Although the citizen scientists participated in additional aspects of this study that are not reported here, they spent about 7 hours over a 6-month period on the tasks required for this portion of the project. For the full study, the citizen scientists received payments of $166.75 by check every 3 months for their participation in the full research project over a 9-month to one-year time period. The portion of their compensation that can be linked specifically to their time on this project is a prorated amount of $47. The timeline for data collection, training, radon testing, report back, and the focus group is shown in Figure 2.

### CITIZEN SCIENCE TRAINING

The 2-hour virtual citizen science training included an overview of the study goals; the role of the citizen scientist as a member of the study team; details of their participation; an introduction to radon, home radon testing and mitigation; and detailed instructions for using the Airthings® Corentium Home Radon Detector and for reporting of daily and 2-week long-term values. In preparation for the training, we mailed each participant an Airthings® Corentium Home Radon Detector and detector instruction sheet developed by the study team. First, the PI welcomed the citizen scientists to the Radon on the RADAR study and introduced the RADAR team members. The Project Manager then provided an overview of the study goals and reviewed the role of citizen scientists as study team members, and reviewed participation guidelines. Second, we provided the citizen scientists with education on the basics of radon including the characteristics and sources of radon; a review of geology and its role in radon risk potential; how radon enters a home; lung cancer risk from radon exposure, particularly among those who are also exposed to tobacco; methods of home radon testing; how to fix high levels of home radon; and regulations for disclosing home radon values during real estate transactions. Lastly, we provided the citizen scientists with instructions on how to choose where to place, and how to use the detector, including how to read the values on the radon detector and report both daily and 2-week long-term average radon values to the study team. At the end of the training, citizen scientists completed a Radon Testing Plan to decide where to place their detector. Table 1 provides a summary of the citizen scientist training content and timeline for each training topic.

### AIRTHINGS® HOME RADON TESTING

The Airthings® Corentium Home Radon Detector provides an immediate and continuous measurement of radon levels within 24 hours (Airthings® 2021). We asked citizen scientists to report daily radon values for 14 days and an average 2-week radon value on day 15. We sent citizen scientists a daily SMS text or email with a survey link to enter their radon values. However, we were unable to offer the SMS option to all citizen scientists due to a technical issue. Nearly four in ten citizen scientists (39%) submitted their radon values using a texted survey link.

### REPORT BACK

Based on the reported 2-week long-term average values, the RADAR team developed a 1-page personalized report for each citizen scientist showing both their daily radon values and the 2-week average. Each 1-page personalized report displayed a line chart with each daily value, depicting horizontal lines for the Environmental Protection Agency (EPA) and World Health Organization (WHO) action levels, and the average 2-week average below the chart, with an interpretive statement, “Your radon value is [at or above or below] the [EPA or WHO] action level of [4.0 or 2.7 pCi/L, respectively].” We attached the personalized 1-page report to a tailored email that discussed their results and recommended next steps. The tailored email included their average 2-week radon level, the interpretive statement, a reminder about how radon is measured, how to fix high radon as appropriate, and additional resources about radon and tobacco smoke from national and state websites. If the citizen scientist reported smoking in the home at baseline, we also provided resources on secondhand smoke and tobacco use cessation.

For those with 2-week average levels at or above the WHO action level (but below the EPA action level), we included in the personalized email a recommendation to test for an additional two months (and how to reset the detector to take a longer-term reading). We invited those with 2-week long-term average radon values at or above the EPA action level to discuss their radon values and benefits of mitigation via telephone or Zoom, using a 20-minute brief problem-solving approach to assess readiness to take action, including the participant’s perceived risk, worry, social norms (“how concerned about radon are people in your community”), response efficacy (“do you know anyone who has mitigated”), and barriers to mitigation (e.g., cost) adapted from a previous lung cancer risk–reduction study (Huntington-Moskos et al. 2021). Two members of the RADAR team led the informal conversation with the participant to answer questions and describe the mitigation process, how to contact a certified radon mitigation professional, and how to use the study voucher to partially cover the cost. Following the conversations, we sent all participants with radon values at or above the EPA action level a personalized mitigation voucher worth 30% of the cost up to $600 when using a study-approved certified radon mitigation professional. Each of the vouchers included a list of 2 to 3 certified radon mitigation professionals, and the email provided instructions on how to redeem the voucher when pursuing mitigation. Citizen scientists with high radon levels were encouraged but not required to mitigate for radon.

### FOCUS GROUP

Lastly, we invited all citizen scientists to participate in one of ten 1-hour focus groups, during which we assessed their experience with home radon testing and gathered their thoughts on the best ways to communicate radon risk with community members. We used a semi-structured interview guide to prompt informal discussion of the training sessions, on using the Airthings® device, on reporting radon values, and on how we might promote broader access to the radon devices in their community (e.g., libraries, effective messaging).

### DATA COLLECTION

Participants were invited to complete the online survey evaluating EHL and efficacy measures at baseline 2-days after the radon testing period and again approximately 4–5 months post-testing. All participant surveys were developed using Qualtrics^©^ (Qualtrics Software 2021), an online survey and data accumulation platform. Daily, during the 2-week testing period, participants were invited via email or text message to complete an online survey to report their 1-day radon average. At the end of the 2-week testing period, they were asked to report the 2-week long-term average radon value using the same method.

### MEASURES

Demographics including sex, race/ethnicity, age, household income, education, an indicator for whether any household members used tobacco (including e-cigarettes), and an indicator for whether there was a family history of lung cancer were collected at baseline. Each of the three participant surveys included measures of EHL, response efficacy, health information efficacy, and self-efficacy. The 14-item EHL measure assesses knowledge of radon exposure as a health hazard at each level of the Finn and O’Fallon (2017) EHL framework, guiding measurement of knowledge ranging from recognizing an environmental health threat to creating strategies to prevent or mitigate exposure based on an adapted version of Bloom’s Taxonomy (Bloom 1956). The EHL measure assessed respondents’ ability to: 1) recognize radon exposure as a potential health hazard; 2) understand the ways in which radon exposure occurs (e.g., cracks in foundation or other contact with soil [Stone, Uesugi, and Hirsch 2012]); 3) apply that understanding to inform preventive action such as home radon testing; 4) analyze data to assess personal radon exposure risk; 5) evaluate radon mitigation options; and 6) create a radon exposure mitigation plan. Scores range from 0 to 14, with higher scores reflecting greater EHL related to radon as an environmental health threat, and the benefits of testing and mitigation strategies.

The 3-item response efficacy scale uses a 5-point Likert scale (strongly disagree [0] to strongly agree [4]) to measure the belief that radon testing can identify health threats and provide opportunities for reducing disease risk (Witte 1992; Witte et al. 1998; Wong 2009). Scores range from 0 to 12, with higher scores reflecting greater perceived response efficacy related to radon testing. The 2-item health information efficacy scale uses a 5-point Likert scale (strongly disagree [0] to strongly agree [4]) to measure the ability to search and process radon information (Basu and Dutta 2008). Scores range from 0 to 8, with higher scores reflecting greater ability to search and process radon information. Lastly, the 3-item self-efficacy measures assess confidence in the respondent’s ability to take each of three health-protective actions: home radon testing, contacting a radon professional, and hiring a radon professional (Bandura 1977; Witte 1992; Witte et al. 1996). The 3-items measure ability, resources, and ease of action using a 5-point Likert scale (strongly disagree [0] to strongly agree [4]). Separate self-efficacy scores are determined for each health action with scores ranging from 0 to 12, with higher scores reflecting greater confidence in the respondent’s ability to test for radon, contact a radon professional, and hire a radon professional.

### DATA ANALYSIS

The data were summarized using descriptive statistics, including means and standard deviations or frequency distributions. Mixed modeling for repeated measures was used to evaluate changes over time, with separate models for EHL and each subset of response efficacy, health information efficacy, and self-efficacy. In each model, the main effect was time (baseline, post-testing, 4–5 months); post-hoc analysis of significant time effects was accomplished using Fisher’s least significant difference procedure. Participant education and indicators for high radon at baseline, family history of lung cancer, and one or more tobacco users living in the home were included in each model as covariates to adjust for differences in outcomes related to these demographic characteristics. We used SAS, v. 9.4 (SAS Institute Inc. 2016). As a protection against an inflated overall Type I error rate, we used an alpha level of .01 for inferential testing. Data collected from the semi-structured interviews during the focus group were not coded; rather, investigators listened for ways to clarify the instruction guides to inform future home radon testing using Airthings®, and elicited recommendations to inform our team on how best to encourage others in the community to test for radon.

## RESULTS

We examined changes in environmental health literacy (EHL) and efficacy over time. The average age of the 60 citizen scientists was 51.3 years (*SD* = 13.5); they ranged in age from 25 to 78 (see Table 2). Participants were mostly female (70%); consistent with the population in the four study counties, the majority were white, non-Hispanic (86%). Most citizen scientists had some post-secondary education with at most a college degree (53%). The largest percentage had annual household incomes ranging from $45,000 to less than $90,000 (49%). Nearly one-fourth had a family history of lung cancer (23%), and 20% reported that at least one person living in their home smoked cigarettes, cigars or pipes, or used electronic cigarettes. At the end of the 2-week testing period, 27 homeowners (45%) reported an average long-term radon level at or above the EPA action level of 4.0 pCi/L. The mean of the 2-week average radon values across all 60 participants was 7.0 pCi/L (*SD* = 10.1), with values ranging from 0.2 to 42.6 pCi/L.

### PARTICIPATION IN STUDY COMPONENTS

Nearly all of the 60 citizen scientists participated in the three online surveys at baseline, post-testing, and follow up (100%, 100%, and 97%, respectively). All 60 participants completed the citizen science training. All completed the 2-week radon testing period, with 96% of the 900 expected daily and 2-week radon values reported. Of the 27 participants with 2-week average radon values at or above 4.0 pCi/L, 14 (52%) participated in the informal, conversational report back via phone or Zoom. As of October 2021, 4 of the 27 participants with high radon reported mitigating for radon (15%). Of the 60 citizen scientists, 49 (82%) participated in one of the ten focus groups.

### REPEATED MEASURES MODELING

The repeated measures mixed models to evaluate changes over time in the study outcomes (controlling for whether the participant had high radon at baseline, as well as education, family history of lung cancer, and whether there were tobacco user(s) in the home) demonstrated significant time effects for EHL, health information efficacy, and self-efficacy to test for radon and contact a radon mitigation professional (see Table 3). For EHL, the participant mean score at Time 1 was 8.9, and this increased to 11.4 at Time 2 and 11.9 at Time 3. The post-hoc comparisons indicated that EHL increased significantly from Time 1 to Time 2 and from Time 2 to Time 3. For health information efficacy, while there was an increase from Time 1 (*M* = 5.1) to Time 2 (*M* = 6.0) to Time 3 (*M* = 6.2); the post-hoc analysis revealed that the increase from Time 1 to Time 2 was significant, and it was maintained at Time 3, with no difference in average ability to search and process radon information between Times 2 and 3.

Self-efficacy to test for radon exhibited a pattern similar to that of health information efficacy: There was a significant increase from 9.7 at Time 1 to 10.5 at Time 2, and this was maintained at Time 3, with a mean of 10.7. The difference between Times 2 and 3 was not significant for radon testing self-efficacy. For self-efficacy to contact a radon mitigation professional, the increase from Time 1 (*M* = 8.0) to Time 2 (*M* = 8.4) was not significant, but there was a significant increase from Time 2 to Time 3 (*M* = 9.1). The models with response efficacy and self-efficacy to hire a radon mitigation professional as outcomes did not have significant time effects, so no post-hoc comparisons for time differences were considered for these models.

## DISCUSSION

The citizen science approach to home radon testing and report back was an effective method to promote home radon testing and to increase understanding and processing of radon information, as well as confidence in participants’ perceived ability, resources, and ease of action to test for radon and to contact a mitigation professional. All citizen scientists in this study tested their homes for radon, reporting 96% of the expected radon values over the 2-week testing period. In a previous randomized trial, we recruited participants in primary care and pharmacy settings to test their homes for radon using a charcoal-based radon test kit. Participants were randomly assigned to treatment (*n* = 257) or control (*n* = 258) (Butler et al. 2018). The treatment group received free test kits, while those in the control group were invited to request a test kit from the research team at a later date (simulating standard public health practice). Three-quarters (74%) of the treatment group completed home radon testing compared to 22% in the control group (Butler et al. 2018). The fact that we mailed each citizen scientist a free real-time, continuous home radon detector and instruction guide and provided a 2-hour citizen science training likely boosted the radon testing rate in the study reported here. While the earlier randomized trial (Butler et al. 2018) differs from the current study in that we did not have the same level of participant engagement or training in the former compared to the latter, these results demonstrate the impact of reinforcing participant involvement through a citizen science approach.

The significant increases in environmental health literacy (EHL) over time may suggest that each study component (e.g., citizen science training, real-time home radon testing and daily data entry, report back, and focus group) could have contributed to citizen scientists’ understanding of the connection between radon exposure and human health. However, given the non-experimental study design we cannot determine which component or components may have contributed to increases in EHL. Findings from this study are promising because use of a citizen science approach promoted EHL and led to a high rate of home radon testing. Indeed, several studies have found radon knowledge to be associated with completion of home radon testing (Wang et al. 2000; Duckworth et al. 2002). Novel participatory methods, including citizen science, are needed to increase home radon testing and mitigation and improve EHL.

The citizen science approach to radon testing and report back also improved participants’ perceived ability to search and process radon information in the short-term, and this improvement was maintained over time. Similarly, participants reported a boost in their confidence to test their homes for radon following the citizen scientist training and home radon testing; this testing self-efficacy increase was maintained over time. Interestingly, citizen scientists reported greater confidence to contact a radon mitigation professional only after they received report back and participated in the focus group. Self-efficacy to contact a radon professional did not change after citizen science training and radon testing alone; rather, there was a delayed effect on confidence to contact a radon professional. Perhaps the personalized report back of daily values and average 2-week radon value, along with the tailored email with next steps and phone or zoom conversations with at-risk participants, provided the citizen scientists with the resources needed to boost their confidence in contacting a radon mitigation professional. Indeed, those with high radon levels received a mitigation voucher containing actual names and contact information for radon mitigation professionals serving their county as part of the personalized report back.

Despite our efforts to educate, train, and support the citizen scientists related to hazard remediation, in the case of radon mitigation, we saw no change in their beliefs that mitigation would avoid the health threat from radon exposure (response efficacy), nor in their capacity to hire a radon mitigation professional (mitigation hiring self-efficacy). It is possible that those who do not view themselves at risk for an environmental exposure (e.g., those with 2-week radon values below the EPA action level of 4.0 pCi/L) may answer these questions differently than those at risk (e.g., those with 2-week radon values at or above the EPA action level). Given that citizen scientists were more confident about contacting a radon professional at the follow up period, there was an impact on the first step needed for radon remediation. However, confidence in hiring a radon mitigation professional may be related to concerns about cost of mitigation. Despite providing a voucher for 30% of the cost up to $600, cost of radon mitigation remains a barrier (Hahn et al. 2019). Further research is needed to better understand how the citizen science approach might impact beliefs and actions related to radon mitigation.

This study had several limitations. First, we selected a convenience sample of non-scientists to participate in this citizen science project. As such, selection bias is a limitation to study findings. Second, the financial incentives, while tied to the amount of time required for training, home testing, taking surveys, and participating in a focus group, may have affected the completion rate for each of the components. This latter concern is somewhat mitigated by the need for trained citizen scientists to report back the study findings to others living in their county, so that overall testing for radon (in this case) may be elevated in the larger population. Third, the intensive citizen scientist contact may be impractical and labor-intensive for health and government organizations to implement. However, the annual costs of lung cancer (estimated to be in the hundreds of thousands of dollars) may offset the health and governmental organizations’ costs to implement the level of training that is a key component of the citizen science approach (Chiavacci et al. 2020). Radon testing (and mitigation when indicated) is an environmental justice issue in that those without financial means are often unable to access testing and fix their homes when high levels of radon are found. This is a problem that may be addressed through citizen science, especially with low-income populations, as the citizen non-scientists gain awareness of the problem, and they may be more motivated to help find solutions (see Odunitan-Wayas et al. 2020). Lastly, we recognize that while the citizen science approach to radon testing shows promise in promoting environmental health literacy, efficacy, and radon testing, policy change (e.g., requiring radon testing as part of the real estate transaction) is an important population-level factor in prompting action to protect human health from environmental hazards. Future research is needed to evaluate the impact of policy change, in addition to citizen science approaches, on radon testing.

## CONCLUSION

Citizen science holds promise for reducing environmental risks for lung cancer. Although radon exposure, when combined with tobacco smoke, is a major cause of lung cancer, few people test for radon in their homes. Standard public health practice in the US is for residents to request charcoal-based radon test kits from local health departments or purchase them from home improvement stores. This standard approach has yielded low testing rates. The findings of this novel citizen science approach to radon testing reveal that all citizen scientists tested their homes for radon when they had ready access to real-time electronic detectors. Further, training citizen scientists to join a research team and test their homes, using personalized report back of the radon findings, and engaging them in a focus group boosted environmental health literacy and their perceived ability to search for and process radon information. This citizen science approach also improved confidence in their capacity to test their home for radon and contact a radon mitigation professional. Further research is needed to understand the role of citizen science in timely contact of radon mitigation professionals and remediation of high radon levels.

## Figures and Tables

**Table 1:** Citizen Science Training.

TRAINING CONTENT	LENGTH OF TIME IN MINUTES
Introduction to the RADAR team members	2
Overview of the study goals	2
Role of the citizen scientist as a member of the study team	3
Study participation guidelines	3
Introduction to radon, radon testing & mitigation What is radon and where does it come from? Review of geology and radon potential How does radon get into a home? Health risks from radon Tobacco and radon synergism Radon testing Radon mitigation Disclosure of radon during a real estate transaction	40
Break	10
Use of Airthings Corentium Home Radon Detector Where to test in the home How to start the Airthings detector How to read the numbers on the screen	30
Review schedule of 2-week testing period	3
How to report daily and 2-week long-term radon values	15
Review recommended action, including use of study mitigation voucher, if home tests ≥ 4.0 pCi/L	2
Q&A	10
**Total time**	**120**

**Table 2:** Demographic, personal and home characteristics of the citizen scientist participants (*N* = 60).

CHARACTERISTIC	MEAN (SD) OR N (%)
Age	51.3 (13.5)
Gender	
Male	18 (30.5%)
Female	41 (69.5%)
Race/ethnicity	
White, non-Hispanic	51 (86.4%)
Black or African American	7 (11.9%)
More than one race/ethnicity	1 (1.7%)
Education	
High School/GED	5 (8.3%)
At least some post-secondary (college/vocational)	32 (53.4%)
Postgraduate education	23 (38.3%)
Annual household income	
<$45,000	11 (19.3%)
$45,000 – < $90,000	28 (49.1%)
$90,000 and above	18 (31.6%)
Family history of lung cancer	
Yes	14 (23.3%)
No	46 (76.7%)
Any tobacco users of cigarettes, cigars, or pipes in the home, including participant	
Yes	9 (15.0%)
No	51 (85.0%)
Average radon level in home, during 2-week testing	
Below the EPA action level of 4 pCi/L	33 (55.0%)
At or above the EPA action level of 4 pCi/L	27 (45.0%)
Radon level, averaged over the 2-wk testing period	7.0 (10.1)

**Table 3:** Summary of repeated measures mixed models, including means and standard deviations at each timepoint, F tests for the time main effect, and post-hoc testing (*N* = 60).

OUTCOME (POTENTIAL RANGE)	MEANS AND STANDARD DEVIATIONS AT EACH SURVEY COMPLETION POINT			F TEST AND (P) FOR TIMEPOINT AND RESULTS OF POST-HOC TESTING*
	*BASELINE (TIME 1)*	*POST-TESTING (TIME 2)*	*4–5 MONTHS AFTER POST-TESTING (TIME 3)*	
Environmental health literacy (0–14)	8.9 (0.9)	11.4 (1.0)	11.9 (1.0)	184.8 (<.001) 1 < 2 < 3
Response efficacy (0–12)	9.9 (1.5)	10.3 (1.5)	10.2 (1.6)	1.6 (.22) n/a
Health information efficacy (0 – 8)	5.1 (1.4)	6.0 (1.6)	6.2 (1.5)	14.0 (<.001) 1 < 2, 3
Self-efficacy to test for radon (0–12)	9.7 (1.6)	10.5 (2.0)	10.7 (1.5)	12.0 (< .001) 1 < 2, 3
Self-efficacy to contact radon mitigation pro (0–12)	8.0 (2.2)	8.4 (1.8)	9.1 (2.0)	6.1 (.004) 1, 2 < 3
Self-efficacy to hire radon mitigation pro (0–12)	7.0 (2.8)	7.0 (2.4)	7.6 (2.9)	1.6 (.22) n/a

*Notes*: For each outcome, a higher score indicates greater literacy/ efficacy; education and the indicators for high radon at baseline, family history of lung cancer, and tobacco users in the home were included as covariates in each model.

* Pairwise comparisons significant at alpha < .01 are noted.

## Data Availability

Data used in this research project involves human participants. To maintain anonymity and confidentiality, in accordance with the informed consent obtained from the study participants, the data cannot be made publicly accessible.
